# A double-blind, randomized placebo-controlled trial examining the effect of MitoQ on myocardial energetics in patients with dilated cardiomyopathy

**DOI:** 10.1093/ehjci/jeaf310

**Published:** 2025-11-10

**Authors:** Brian P Halliday, Ruth Owen, Aaraby Ragavan, Katherine L Smith, Ben Statton, Alaine Berry, Alex Kasiakogias, Zoi Tsoumani, Mayooran Shanmuganathan, Jason N Dungu, Antonio de Marvao, Upasana Tayal, James S Ware, Declan P O’Regan, Dudley J Pennell, John G F Cleland, Sanjay K Prasad, John Gregson, Michael P Murphy, Oliver J Rider, Ladislav Valkovič

**Affiliations:** National Heart and Lung Institute, Imperial College London, Sydney Street, London SW3 6NP, UK; Royal Brompton and Harefield Hospitals, Part of Guy’s and St Thomas’ NHS Foundation Trust, London, UK; Department of Medical Statistics, London School of Hygiene and Tropical Medicine, London, UK; Centro Nacional de Investigaciones Cardiovasculares, Madrid, Spain; Oxon Epidemiology, Madrid, Spain; National Heart and Lung Institute, Imperial College London, Sydney Street, London SW3 6NP, UK; Royal Brompton and Harefield Hospitals, Part of Guy’s and St Thomas’ NHS Foundation Trust, London, UK; National Heart and Lung Institute, Imperial College London, Sydney Street, London SW3 6NP, UK; Royal Brompton and Harefield Hospitals, Part of Guy’s and St Thomas’ NHS Foundation Trust, London, UK; Medical Research Council Laboratory of Medical Sciences, Imperial College London, London, UK; Medical Research Council Laboratory of Medical Sciences, Imperial College London, London, UK; Royal Brompton and Harefield Hospitals, Part of Guy’s and St Thomas’ NHS Foundation Trust, London, UK; Royal Brompton and Harefield Hospitals, Part of Guy’s and St Thomas’ NHS Foundation Trust, London, UK; Royal Brompton and Harefield Hospitals, Part of Guy’s and St Thomas’ NHS Foundation Trust, London, UK; Oxford Centre for Clinical MR Research (OCMR), Radcliffe Department of Medicine, University of Oxford, Oxford, UK; Department of Cardiology, Basildon and Thurrock Hospitals NHS Foundation Trust, Basildon, Essex, UK; Anglia Ruskin University, Essex, UK; Department of Cardiology, Guy’s and St Thomas’’ NHS Foundation Trust, London, UK; School of Cardiovascular Science, King’s College London, London, UK; National Heart and Lung Institute, Imperial College London, Sydney Street, London SW3 6NP, UK; Royal Brompton and Harefield Hospitals, Part of Guy’s and St Thomas’ NHS Foundation Trust, London, UK; National Heart and Lung Institute, Imperial College London, Sydney Street, London SW3 6NP, UK; Royal Brompton and Harefield Hospitals, Part of Guy’s and St Thomas’ NHS Foundation Trust, London, UK; Medical Research Council Laboratory of Medical Sciences, Imperial College London, London, UK; Medical Research Council Laboratory of Medical Sciences, Imperial College London, London, UK; National Heart and Lung Institute, Imperial College London, Sydney Street, London SW3 6NP, UK; Royal Brompton and Harefield Hospitals, Part of Guy’s and St Thomas’ NHS Foundation Trust, London, UK; School of Cardiovascular and Metabolic Health, University of Glasgow, Glasgow, UK; National Heart and Lung Institute, Imperial College London, Sydney Street, London SW3 6NP, UK; Royal Brompton and Harefield Hospitals, Part of Guy’s and St Thomas’ NHS Foundation Trust, London, UK; Department of Medical Statistics, London School of Hygiene and Tropical Medicine, London, UK; MRC Mitochondrial Biology Unit, University of Cambridge, Cambridge, UK; Department of Medicine, University of Cambridge, Cambridge, UK; Oxford Centre for Clinical MR Research (OCMR), Radcliffe Department of Medicine, University of Oxford, Oxford, UK; Oxford Centre for Clinical MR Research (OCMR), Radcliffe Department of Medicine, University of Oxford, Oxford, UK; Department of Imaging Methods, Institute of Measurement Science, Slovak Academy of Sciences, Bratislava, Slovakia

**Keywords:** dilated cardiomyopathy, mitochondrial dysfunction, oxidative stress, heart failure

Oxidative stress may be an important driver of dilated cardiomyopathy (DCM) causing mitochondrial dysfunction and reduced ATP production, which mitochondria-targeted antioxidants, such as MitoQ may improve.^1^ We investigated whether MitoQ had an effect on myocardial energetics in patients with DCM using ^31^phosphorus magnetic resonance spectroscopy (^31^P-MRS) in a phase-2 randomized, double-blind, placebo-controlled trial.

Participants provided written informed consent. The trial was approved by the National Research Ethics Committee (21/LO/0035) and registered on ClinicalTrials.gov (NCT05410873). Inclusion criteria were 1) patients with DCM, 2) LVEF ≤45% on two studies ≥3 months apart, 3) on guideline therapy for ≥3 months, 4) in sinus rhythm, 5) elevated NT-pro-BNP (>250 ng/L if >65 years, > 100 ng/L if ≤65 years). Exclusion criteria were 1) atrial fibrillation, 2) contraindication to CMR or gadolinium, 3) environmental trigger for DCM, 4) late gadolinium enhancement (LGE) > 25% 5) current cancer, 6) CoQ10 use and 7) cardiac device.

Participants underwent CMR and ^31^P-MRS on a 3-Tesla scanner (Prisma, Siemens, Germany). Cardiac ^31^P-MRS was performed using a 20 cm loop transmit-receive coil (Pulse Teq, UK) and a 3-dimensional chemical shift imaging sequence.^2^ For skeletal muscle energetics, dynamic ^31^P-MRS was performed with a 15 cm loop transmit-receive coil (Pulse Teq, UK) over the vastus lateralis. With weights on the ankle, participants performed leg raises during two periods of 1 min exercise. This was followed by cine imaging, LGE imaging and extracellular volume (ECV) mapping. NT-pro-BNP was measured (Roche, Switzerland). Patients completed the Kansas City Cardiomyopathy Questionnaire (KCCQ) and a 6-minute walk test.^3^

Patients were randomized 1:1 stratified by left ventricular end-systolic volume (LVESV). Identical MitoQ or placebo were dispensed according to randomization code. Trial staff remained blind to allocation. Participants took two 20 mg capsules of MitoQ or placebo daily and had repeat testing at 3 and 12 months (^31^P-MRS only at 3 months).

The primary endpoint was change in myocardial PCr/ATP at 3 months. Secondary endpoints included change in (i) PCr recovery rate time constant in skeletal muscle, (ii) LVESVi, (iii) LVEF, (iv) ECV, (v) NT-pro-BNP, (vi) 6MWT distance and (vii) KCCQ score. ^31^P-MRS and CMR data were analysed in a standardized fashion by expert operators blind to allocation (OXSA, Oxford, UK^4^; CMR42 V6.0.3, Circle Cardiovascular Imaging, Canada). 34 patients had 90% power to detect a difference of 0.28 in change of PCr/ATP, assuming a standard deviation of 0.25, a 2-sided alpha of 0.05 and a 10% drop-out. The effect of MitoQ was assessed using analysis of covariance on Stata V18.0 (StataCorp, TX, USA). Analyses were by modified intention to treat. *P* < 0.05 was taken as significant.

Of 34 eligible patients, 16 were randomized to MitoQ and 18 to placebo (*Figure 1A)*. One withdrew; another had incomplete primary end-point data; 32 participants were included in the primary analysis. Patients randomized to MitoQ were older with higher NT-pro-BNP but less symptomatic (*Figure 1A*). Rates of heart failure therapy were high.

**Figure 1:**
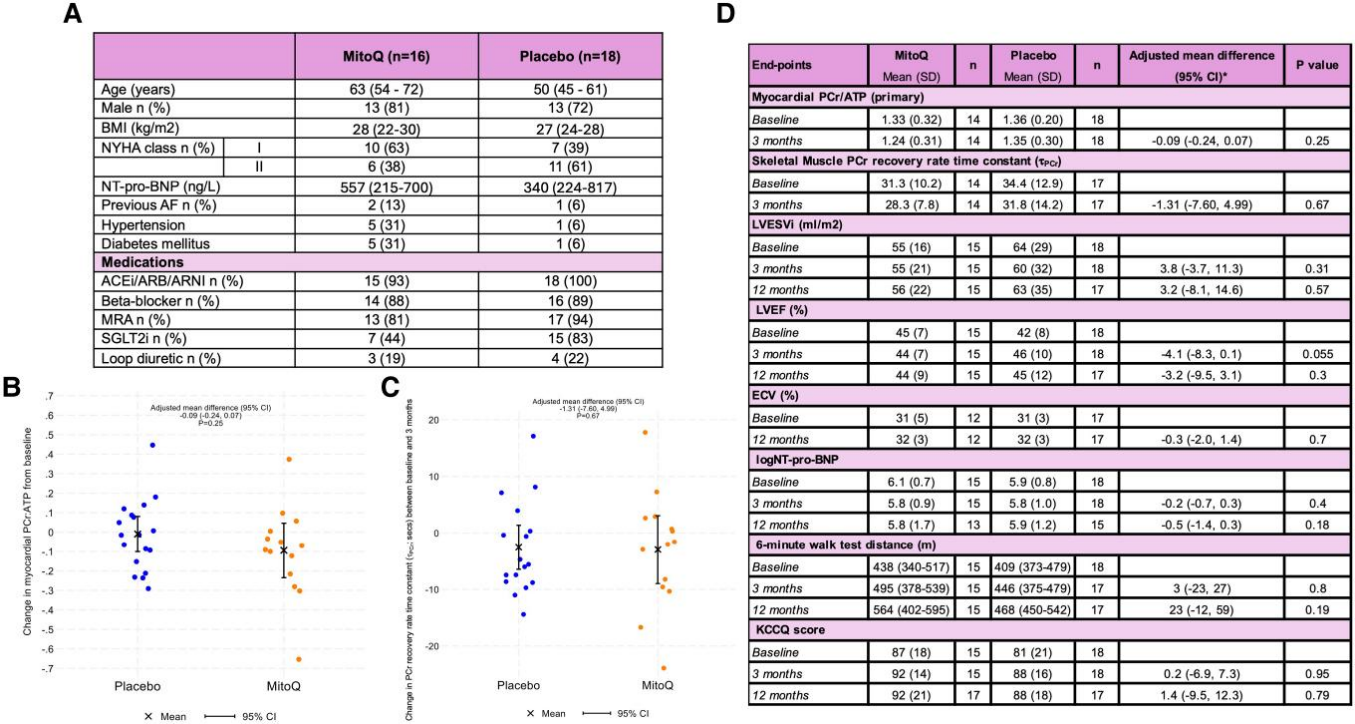
(*A*) Baseline characteristics by randomized treatment allocation; (*B&C*) Change in myocardial and skeletal muscle energetics by treatment allocation; (*D*) Change in primary and secondary end-points by treatment allocation. *Adjusted for baseline value and estimated using analysis of covariance. All differences are summarized as mean (95% CI) except for 6MWT which is summarized as median (95% CI). **6MWT is summarized as median (interquartile range) and *P*-values calculated using the non-parametric Mann-Whitney U test.

After three months, there was no evidence that MitoQ had an effect on myocardial energetics [adjusted mean difference PCr/ATP −0.09 (95% CI −0.24, 0.07); *P* = 0.27] or skeletal muscle energetics (adjusted mean difference PCr recovery rate time constant (τ_PCr_) −1.3 [95% CI −7.6 to 4.9] seconds; *P* = 0.67) (*Figure 1B/1c)*. In addition, MitoQ did not affect LVESV, LVEF, ECV, NT-pro-BNP, 6MWT or KCCQ at 3 or 12 months (*Figure 1D*).

Treatment was well tolerated and self-reported compliance was excellent [mean number of missed doses of 0.5 (1.3)]. Fewer adverse events were reported in the MitoQ arm compared to the placebo arm (26 vs. 49); 3 in the MitoQ and 2 in the placebo arm were at least possibly related to treatment; all graded as mild. There were 3 serious adverse events unrelated to treatment, including a heart failure hospitalization in the MitoQ arm and hospitalizations for pulmonary embolism and kidney injury in the placebo arm.

This trial provides the first randomized data examining the effect of MitoQ on energetics in DCM. We determine that it is unlikely that MitoQ has an effect on myocardial energetics. Our analysis achieved >90% power to detect an improvement in PCr/ATP of at least 0.25; an effect observed with perhexilene.^5^ The upper limit of the 95% confidence interval suggests the chance of a clinically significant effect on energetics is very small. The results may therefore reflect that: (i) mitochondrial oxidative stress is not an important mediator of energetic impairment in DCM or (ii) an effect is only present in a sub-group of patients (such as those with severe heart failure). Altered substrate utilization is an alternative mediator of energetic impairment.^5^

In conclusion, our trial in patients with DCM found that it is unlikely that MitoQ has a clinically significant effect on myocardial energetics. Further work is required to understand energetic dysfunction across the spectrum of DCM, discover the most appropriate therapies and define sub-groups in which they may be most effective.

## Data Availability

Anonymized participant data, the study protocol, including the statistical analysis plan, and a data dictionary are available from the time of publication. Appropriate institutional data transfer agreements will be required. Requests should be made via email to the corresponding author along with an analysis proposal.
